# Supplementary material for: Cited4 is a sex‐biased mediator of the antidiabetic glitazone response in adipocyte progenitors
Source: EMBO Mol Med. 2018 Jul 4;10(8):e8613. doi: 10.15252/emmm.201708613 (PMC6079535; doi:10.15252/emmm.201708613)
Supplement: Supplementary file 1 — Appendix [file EMMM-10-e8613-s001.pdf]

**EMM-2017-08613 - Bayindir-Buchhalter et al.**

# Cited4 is a sex-biased mediator of the antidiabetic glitazone response in adipocyte progenitors

## Appendix

Contents

Appendix Figure S1. Targeting strategy and validation of *Cited4* gene inactivation in the mouse.

Appendix Figure S2. Cited4- and Rosi/PPARg-dependent transcriptional programs overlap in differentiating adipocyte progenitors.

Appendix Figure S3. *Cited4* deficiency does not affect fat accumulation and adipocyte number upon rosiglitazone treatment.

Appendix Figure S4. Ucp1 mRNA and protein correlation in female and male mice.

Appendix Figure S5. Sex-specific involvement of *Cited4* in thermogenic expression and insulin sensitization upon therapeutic rosiglitazone treatment.

Appendix Table S1. Top 15 most significantly enriched gene sets in the gene fraction downregulated by Cited4 knockout.

Appendix Table S2. Cardiovascular phenotype data for Cited4 knockout mice.

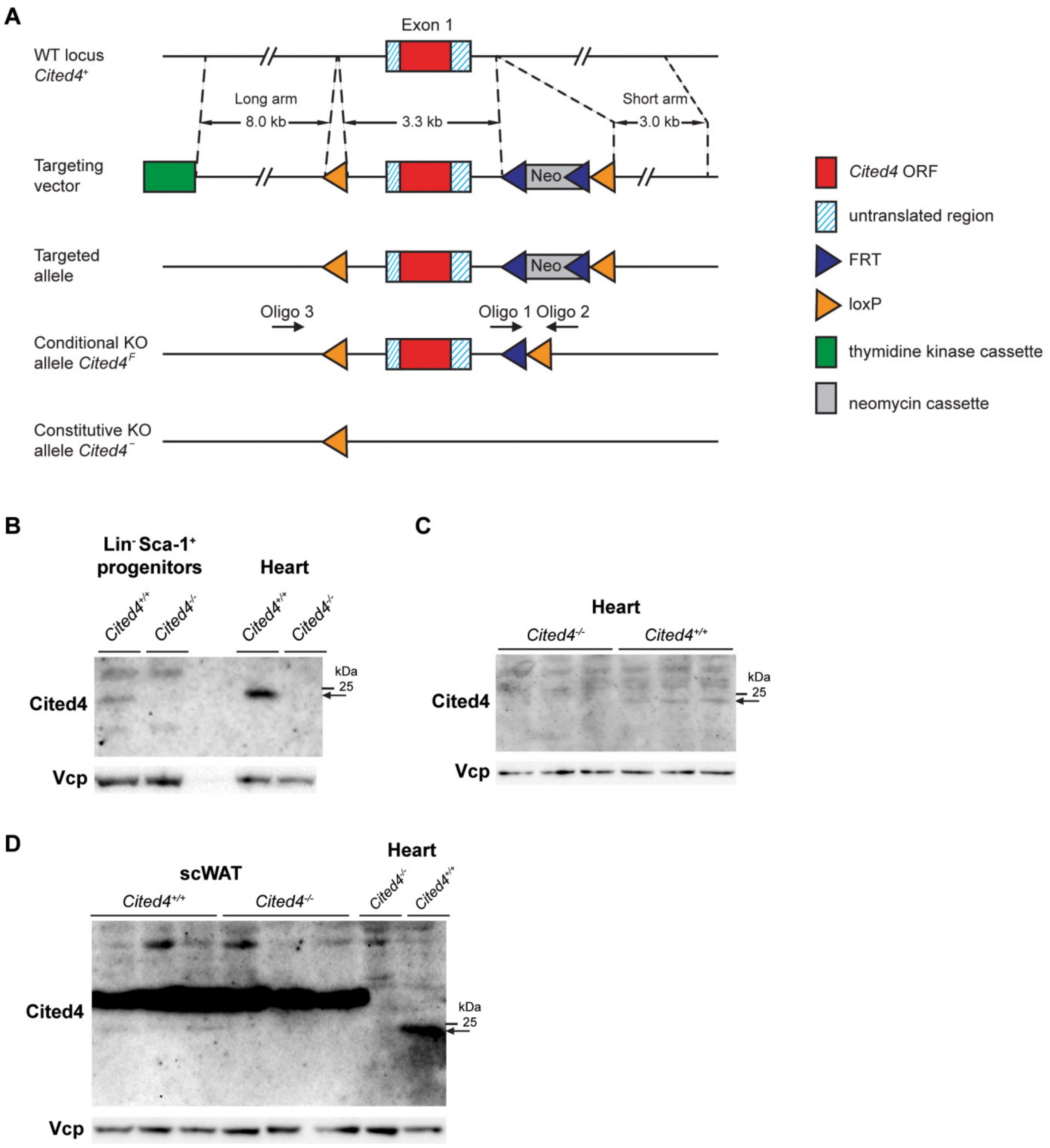


**Appendix Figure S1. Targeting strategy and validation of *Cited4* gene inactivation in the mouse.**

A Targeting strategy for the *Cited4* gene locus (note that *Cited4* is a single-exon gene). Targeting by homologous recombination was performed in ES cells. The *Cited4*$^{F}$ allele was generated by breeding to an FLP recombinase expressing mouse. The *Cited4*$^{-}$ allele was generated by breeding to a Cre recombinase expressing mouse. Oligo 1-3 were used to genotype and distinguish between the *Cited4*$^{+}$, *Cited4*$^{F}$ and *Cited4*$^{-}$ alleles. Map is not to scale.

B-D Cited4 expression in Lin$^{-}$Sca1$^{+}$ progenitors isolated from subcutaneous fat (scWAT), heart and scWAT tissue, as determined by Western blot with Vcp as loading control (lanes represent independent samples, representative of at least 3 biological replicates/mice). Arrow indicates the Cited4 band as predicted at 18 kDa.

**Source data are available online for this figure.**

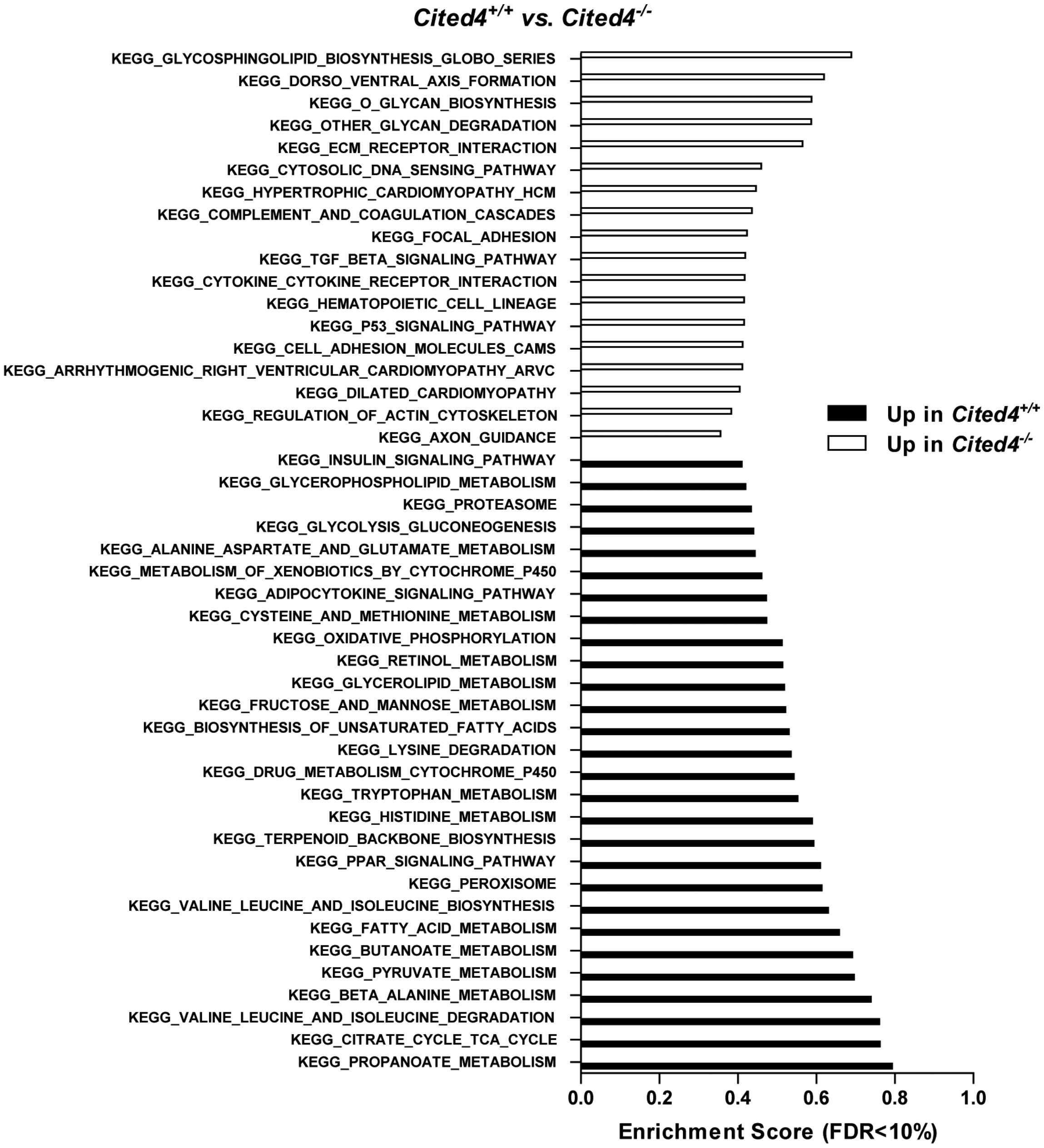


**Appendix Figure S2. Cited4- and Rosi/PPARg-dependent transcriptional programs overlap in differentiating adipocyte progenitors.**

Enrichment scores of gene sets with FDR<0.1 in GSEA performed with the KEGG gene set collection (*Cited4*$^{-/-}$ vs. *Cited4*$^{+/+}$) on expression profiles from female Lin$^{-}$Sca1$^{+}$ progenitors 2 days after induction of differentiation with 100 nM Rosi (n=3).

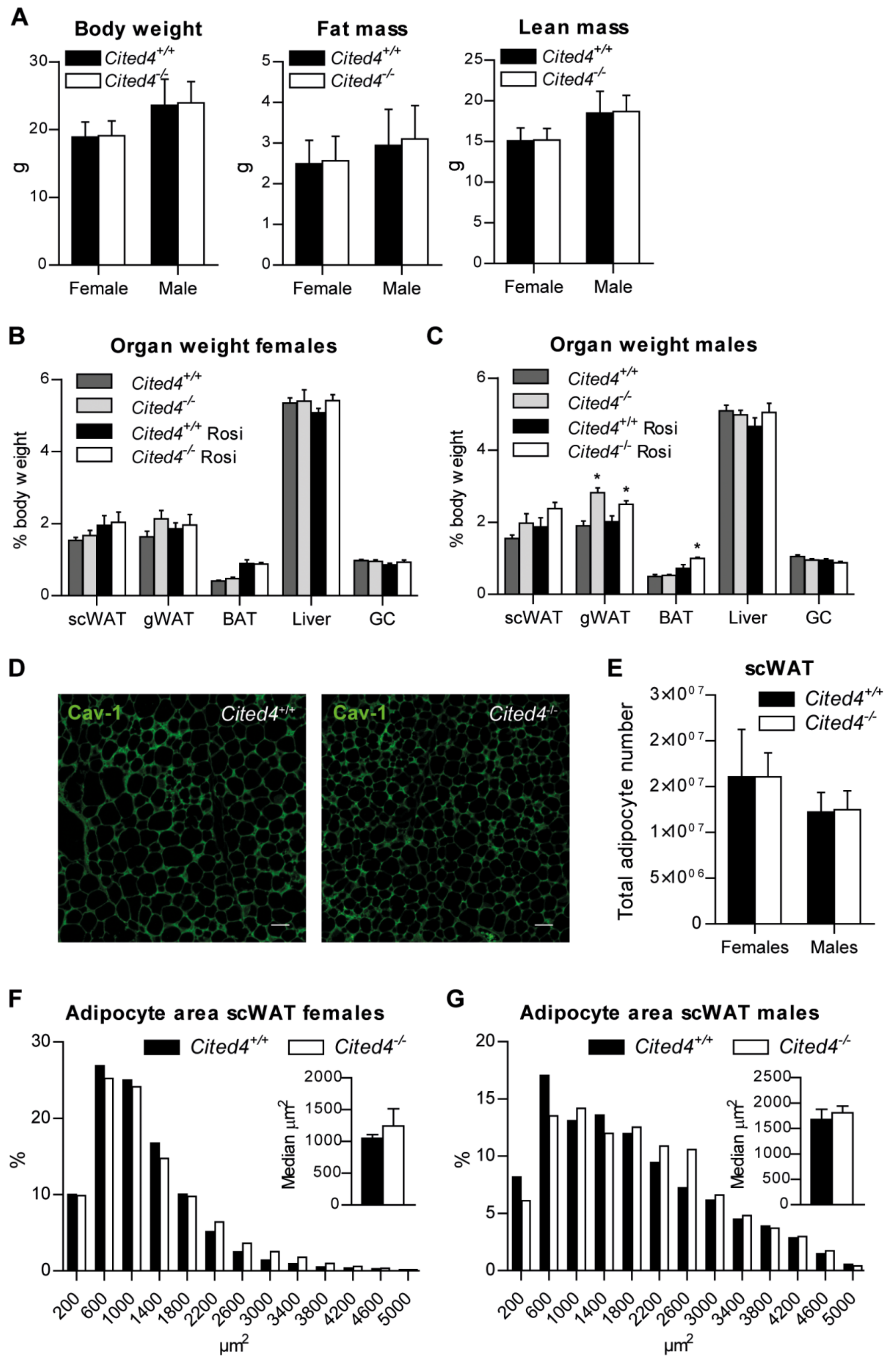


**Appendix Figure S3. *Cited4* deficiency does not affect fat accumulation and adipocyte number upon rosiglitazone treatment.**

A Body composition of mice at 8±2 weeks of age without treatment, determined by NMR quantitation (n=51/48/47/49, *t*-test *Cited4*$^{-/-}$ vs. *Cited4*$^{+/+}$).

B, C Organ weights relative to body weight of mice fed a diet with 0.0075% Rosi or control diet for 2.5 weeks. *t*-test *Cited4*$^{-/-}$ vs. *Cited4*$^{+/+}$ (Rosi), (B) n=5/5/6/6, (C) n=5/4/5/5, *$P$=0.037. GC=Gastrocnemius muscle.

D Caveolin-1 (Cav-1) staining (fluorescence microscopy) for the detection of cell borders in scWAT sections from female mice fed a diet with 0.0075% Rosi or control diet for 2.5 weeks (n=3). Representative images; scale bar is 50 µm.

E Total number of scWAT adipocytes per mouse, calculated based on cell volume quantification on the Cav-1 staining, as in (D) (n=3). *t*-test *Cited4*$^{-/-}$ vs. *Cited4*$^{+/+}$.

F, G Distribution of adipocyte area quantified based on the Cav-1 staining, as in (D) (n=3). Inlet: Average of the distribution medians (n=3), *t*-test *Cited4*$^{-/-}$ vs. *Cited4*$^{+/+}$.

Data information: Data are presented as mean ±SEM unless otherwise indicated and with the exception of (A) showing mean ±SD.

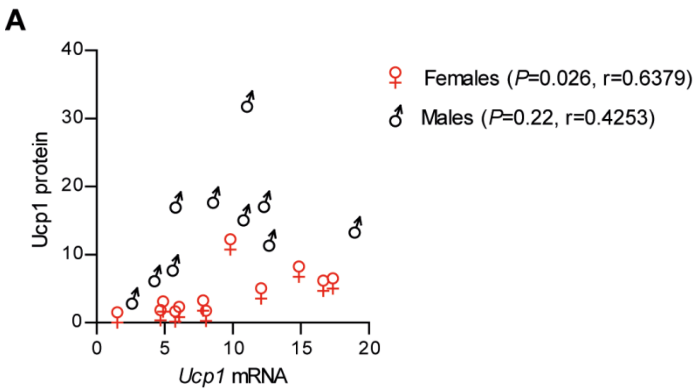


**Appendix Figure S4. Ucp1 mRNA and protein correlation in female and male mice.**

A *Ucp1* mRNA and protein expression in scWAT of mice fed a diet with 0.0075% Rosi or control diet for 2.5 weeks, determined by qRT-PCR and Western blot, respectively. n=12 (females) and n=10 (males). *P*-value and correlation coefficient (*r*) are shown from Pearson correlation analysis.

Data information: Data are presented as individual mice.

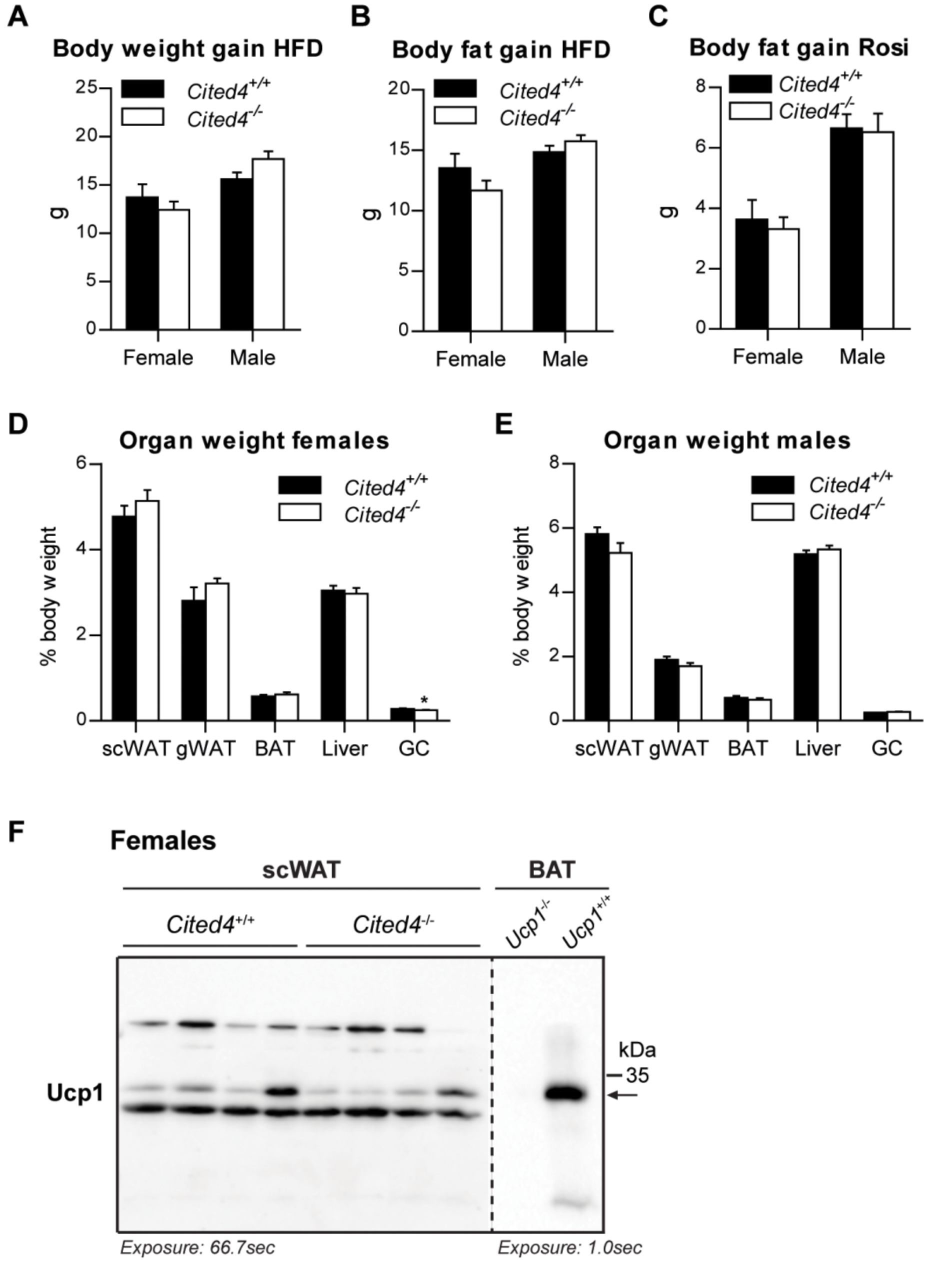


**Appendix Figure S5. Sex-specific involvement of Cited4 in thermogenic expression and insulin sensitization upon therapeutic rosiglitazone treatment.**

A, B Body weight and fat mass gain of mice during 8-9 weeks of HFD (n=16/17/16/16, *t*-test *Cited4*$^{-/-}$ vs. *Cited4*$^{+/+}$).

C Body fat mass gain of mice during 4 weeks of HFD+Rosi treatment, following 16 weeks of HFD (n=16/17/16/16, *t*-test *Cited4*$^{-/-}$ vs. *Cited4*$^{+/+}$).

D, E Organ weights relative to body weight of mice fed HFD for 16 weeks followed by 5 weeks of HFD+Rosi. *t*-test *Cited4*$^{-/-}$ vs. *Cited4*$^{+/+}$, (D) n=8/9, **P*=0.027, (E) n=8/9.

F Ucp1 protein expression in scWAT of mice fed a high-fat diet (HFD) for 11 weeks followed by 5 weeks of HFD with 0.0075% Rosi or in BAT of mice on control diet, determined by Western blot. scWAT and BAT samples were on the same blot but are shown at different exposures. Arrow indicates the band specific to Ucp1. *Ucp1*$^{+/+}$ and *Ucp1*$^{-/-}$ indicate mice homozygous for *Ucp1* wild type and null alleles, respectively..

Data information: Data are presented as mean ±SEM.

**Appendix Table S1. Top 15 most significantly enriched gene sets in the gene fraction downregulated by *Cited4* knockout.**

| Gene set name | Size | Enrichment score[1] | Nominal p-value | FDR q-value |
|---|---|---|---|---|
| V$SF1_Q6 | 231 | -0.34 | 0.002 | 0.130 |
| V$PPARG_01 | 42 | -0.47 | 0.004 | 0.195 |
| ACTAYRNNNCCCR_UNKNOWN | 402 | -0.30 | 0.002 | 0.276 |
| TNCATNTCCYR_UNKNOWN | 117 | -0.34 | 0.006 | 0.287 |
| YAATNANRNNNCAG_UNKNOWN | 57 | -0.39 | 0.006 | 0.363 |
| TGCGCANK_UNKNOWN | 499 | -0.27 | 0.002 | 0.377 |
| V$ROAZ_01 | 10 | -0.53 | 0.157 | 0.417 |
| V$YY1_Q6 | 208 | -0.30 | 0.004 | 0.421 |
| AACYNNNNTTCCS_UNKNOWN | 83 | -0.34 | 0.042 | 0.433 |
| GKCGCNNNNNNNTGAYG_UNKNOWN | 50 | -0.35 | 0.093 | 0.438 |
| TGACCTTG_V$SF1_Q6 | 225 | -0.28 | 0.026 | 0.445 |
| V$PPAR_DR1_Q2 | 238 | -0.28 | 0.014 | 0.453 |
| V$HNF4_DR1_Q3 | 237 | -0.29 | 0.018 | 0.455 |
| GCCATNTTG_V$YY1_Q6 | 375 | -0.27 | 0.015 | 0.465 |
| GGAANCGGAANY_UNKNOWN | 98 | -0.31 | 0.080 | 0.480 |

RNA expression profiling was performed with Affymetrix Gene arrays on *Cited4*$^{-/-}$ and *Cited4*$^{+/+}$ progenitor cells 2 days after differentiation induction with Rosi (n=3). Global expression profiles (*Cited4*$^{-/-}$ vs. *Cited4*$^{+/+}$) were subjected to Gene Set Enrichment Analysis (GSEA) with the TFT gene set collection. Gene sets were ranked by the False Discovery Rate (FDR) q-value reflecting significance.

[1] Maximum of the corresponding enrichment score curve.

**Appendix Table S2. Cardiovascular phenotype data for *Cited4* knockout mice.**

| Parameter | Female | | | Male | | |
|---|---|---|---|---|---|---|
| | ***Cited4***$^{+/+}$ | ***Cited4***$^{-/-}$ | ***t*-test** | ***Cited4***$^{+/+}$ | ***Cited4***$^{-/-}$ | ***t*-test** |
| | (n=5) | (n=10) | *P*-value | (n=9) | (n=7) | *P*-value |
| Systolic pressure (mmHg) | 108.17 ± 6.99 | 113.99 ± 7.34 | 0.165 | 109.51 ± 6.08 | 111.92 ± 5.28 | 0.420 |
| Diastolic pressure (mmHg) | 96.49 ± 7.39 | 102.58 ± 7.78 | 0.170 | 97.25 ± 7.88 | 100.83 ± 5.52 | 0.325 |
| Mean arterial pressure (mmHg) | 100.05 ± 7.15 | 106.06 ± 7.53 | 0.163 | 101.00 ± 7.20 | 104.18 ± 5.23 | 0.344 |
| Pulse (bpm) | 642.11 ± 41.59 | 629.47 ± 32.96 | 0.531 | 611.72 ± 47.95 | 561.21 ± 32.61 | 0.032 |
| Heart weight (mg) | 120 ± 26 | 109 ± 9 | 0.215 | 129 ± 7 | 135 ± 17 | 0.324 |
| Heart weight normalized to tibia length (mg/mm) | 6.7 ± 1.5 | 6.0 ± 0.6 | 0.205 | 6.8 ± 0.3 | 7.3 ± 0.9 | 0.170 |
| Heart weight normalized to body weight (mg/g) | 5.4 ± 1.0 | 4.9 ± 0.5 | 0.216 | 4.3 ± 0.4 | 4.5 ± 0.4 | 0.353 |

Data represent mean ± standard deviation. n refers to the number of mice per group. *t*-test refers to *Cited4*$^{-/-}$ versus *Cited4*$^{+/+}$. Blood pressure and pulse were measured by a tail cuff method on conscious mice and repeated measurements over 4 days were averaged.
