# Supplementary material for: Cited4 is a sex‐biased mediator of the antidiabetic glitazone response in adipocyte progenitors
Source: EMBO Mol Med. 2018 Jul 4;10(8):e8613. doi: 10.15252/emmm.201708613 (PMC6079535; doi:10.15252/emmm.201708613)
Supplement: Supplementary file 2 — Expanded View Figures PDF [file EMMM-10-e8613-s002.pdf]

Expanded View Figures

Figure EV1. *Cited4* is a target of rosiglitazone in murine adipocyte progenitors promoting beige differentiation.

A mRNA expression in C3H10T1/2 cells, differentiated in the presence of 1 μM Rosi or vehicle for the indicated time, as determined by qRT–PCR (day 0, n = 3; day 4, n = 4; day 6, Ctrl, n = 2, Rosi, n = 4; day 10, n = 4). ***P = 0.001, **P = 0.007 (Day 6), **P = 0.005 (Day 10), in 2 × 2 ANOVA with Bonferroni's posttests (Rosi vs. vehicle).

B mRNA expression in 3T3-L1 cells, differentiated in the presence of 1 μM Rosi or vehicle for the indicated time, as determined by qRT–PCR (n = 3). 2 × 2 ANOVA with Bonferroni's posttests, $P > 0.05$ (Rosi vs. vehicle).

C mRNA expression in female $Lin^{-}Sca1^{+}$ cells, differentiated in the presence of 0.1 or 1 μM pioglitazone (Pio) or vehicle for 8 days, as determined by qRT–PCR (n = 4 for Day 2, n = 4/2/6 for Day 8). ***$P = 1*10^{-10}$, **P = 0.003, in 2 × 2 ANOVA with Bonferroni's posttests (Pio vs. vehicle).

D mRNA expression in female $Lin^{-}Sca1^{+}$ cells, differentiated in the presence of the indicated substances for 8 days, as determined by qRT–PCR (n = 3). **P = 0.0035, ***$P = 4 \times 10^{-10}$, ***$P = 7 \times 10^{-10}$ (*Ucp1*), *P = 0.036, ***P = 0.0002, ***$P = 1 \times 10^{-10}$, ***$P = 1 \times 10^{-10}$ (*Cpt1b*), ***$P = 2 \times 10^{-5}$ (*Cidea*), ***$P = 1 \times 10^{-6}$, ***$P = 4 \times 10^{-9}$, ***$P = 1 \times 10^{-8}$, (*Elovl3*), ***$P = 9 \times 10^{-8}$, ***$P = 2 \times 10^{-7}$ (*Cox7a1*), ***P = 0.0002, ***$P = 1 \times 10^{-8}$, ***$P = 4 \times 10^{-5}$, ***P = 0.0002 (*Cox8b*), *P = 0.032, **P = 0.006, ***P = 0.0007 (*Dio2*), ***$P = 1 \times 10^{-6}$, ***$P = 2 \times 10^{-8}$ (*Cyc1*), ***$P = 8 \times 10^{-5}$, ***$P = 3 \times 10^{-6}$ (*Ndufb3*) in 2 × 2 ANOVA with Holm–Sidak posttests ($Cited4^{-/-}$ vs. $Cited4^{+/+}$).

E mRNA expression in male $Lin^{-}Sca1^{+}$ progenitor cells, differentiated in the presence of 100 nM Rosi or vehicle for the indicated time, as determined by qRT–PCR (n = 3). ****$P = 1 \times 10^{-10}$ (Days 2 and 4), ****$P = 4 \times 10^{-8}$ (Day 8) in 2 × 2 ANOVA with Bonferroni's posttests (Rosi vs. vehicle).

F mRNA expression in male $Lin^{-}Sca1^{+}$ cells, differentiated in the presence of 100 nM Rosi or vehicle for 8 days, as determined by qRT–PCR (n = 3, t-test).

Data information: Data are presented as mean ± SEM except for (C) (individual data).

© 2018 The Authors

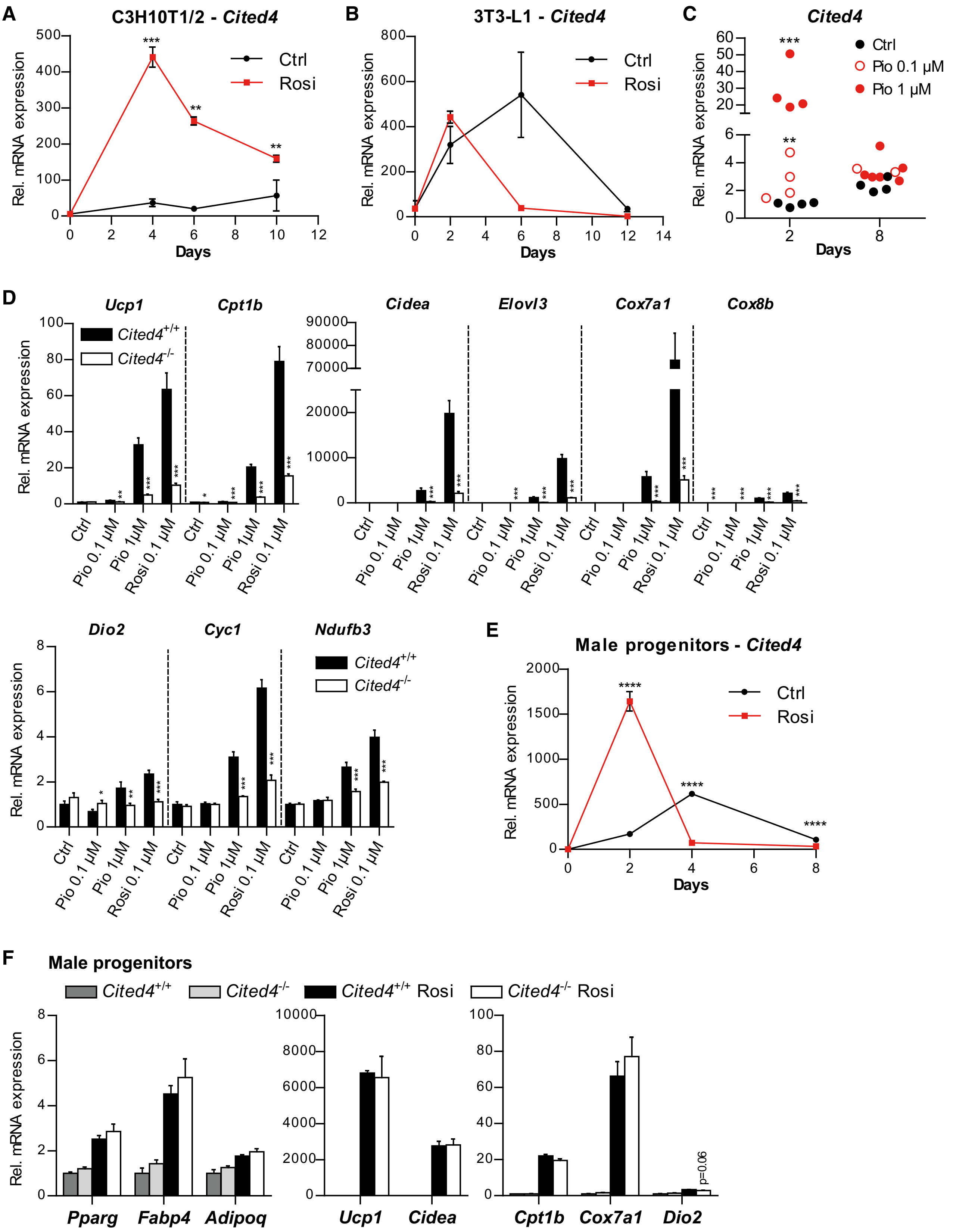

Figure EV1.

 © 2018 The Authors

Figure EV2. *Cited4* is a target of rosiglitazone in murine and human adipocyte progenitors promoting beige differentiation.

A Phase contrast microscopy of primary SVF cells from human subcutaneous fat, differentiated in the presence of 100 nM Rosi or vehicle for 14 days (representative of n = 5 patients). Scale bar is 100 μm.

B, C mRNA expression in primary SVF cells from human subcutaneous fat, differentiated in the presence of 100 nM Rosi or vehicle, as determined by qRT–PCR (n = 5 patients). (B) ****$P = 1 \times 10^{-10}$ (Day 2), ****$P = 5 \times 10^{-8}$ (Day 6), ****$P = 1 \times 10^{-9}$ (Days 10 and 14), (C) ****$P = 7 \times 10^{-7}$ (Day 6), ****$P = 1 \times 10^{-9}$ (Days 10 and 14) in 2 × 2 ANOVA with Bonferroni's posttests (Rosi vs. vehicle).

D mRNA expression in primary SVF cells from human male subcutaneous fat transfected with the indicated siRNA prior to differentiation in the presence of 100 nM Rosi for 9 days, as determined by qRT–PCR (n = 3). ***P = 0.0002 (*siCITED4.1 CITED4*), ***P = 0.0003 (*siCITED4.2 CITED4*), **P = 0.005 (*UCP1*), *P = 0.01 (*CIDEA*), ***P = 0.0006 (*CITED4.1 CPT1B*), **P = 0.007 (*CPT1B*), ***P = 0.0006 (*CITED4.1 SLC2A4*),**P = 0.009 (*ADIPOQ*) in ANOVA with Tukey's posttests (vs. *siCtrl*).

E GFP fluorescence intensity distribution of $Lin^{-}Sca1^{+}$ progenitor cells 24 hours after transfection with GFP mRNA, determined by flow cytometry (compared to non-transfected cells).

F mRNA expression in female $Cited4^{F/F}$ $Lin^{-}Sca1^{+}$ progenitor cells transfected with Cre or control mRNA prior to differentiation in the presence of 100 nM Rosi or vehicle for 8 days, as determined by qRT–PCR (n = 3). t-test Cre vs. Ctrl (Rosi), **P = 0.002 (*Cited4*), *P = 0.039 (*Adipoq*), *P = 0.015 (*Ucp1*), *P = 0.011 (*Cidea*), **P = 0.004 (*Cpt1b*), *P = 0.011 (*Dio2*).

G mRNA expression in female $Cited4^{F/F}$ $Lin^{-}Sca1^{+}$ progenitor cells transfected with Cre or control mRNA 3 days after induction of differentiation in the presence of 100 nM Rosi or vehicle for 8 days, as determined by qRT–PCR (n = 4). t-test Cre vs. Ctrl, ***P = 0.0005 (*Cited4*), **P = 0.009 (*Cpt1b*).

Data information: Data are presented as mean ± SEM except for (E) (individual data).

© 2018 The Authors

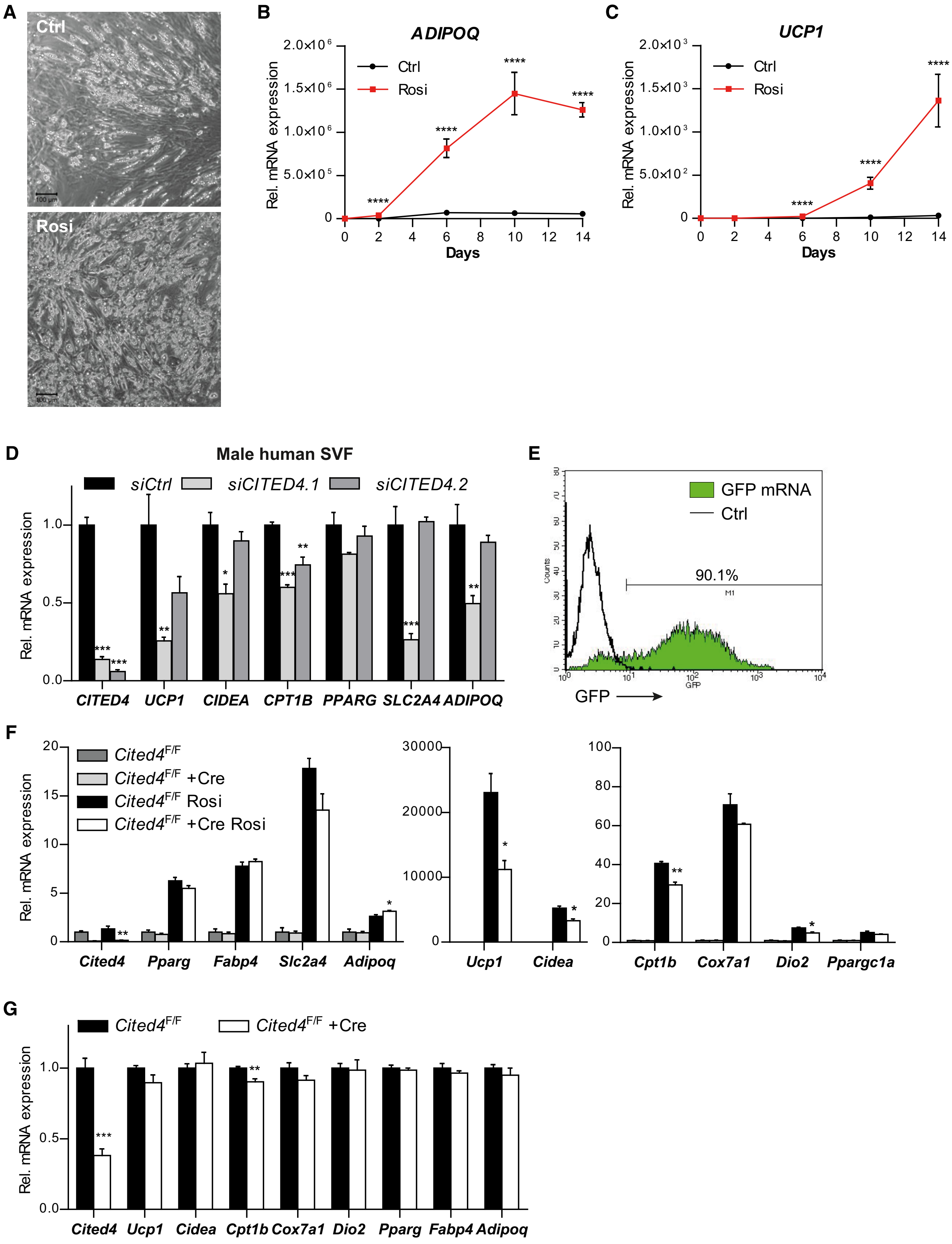

Figure EV2.

© 2018 The Authors

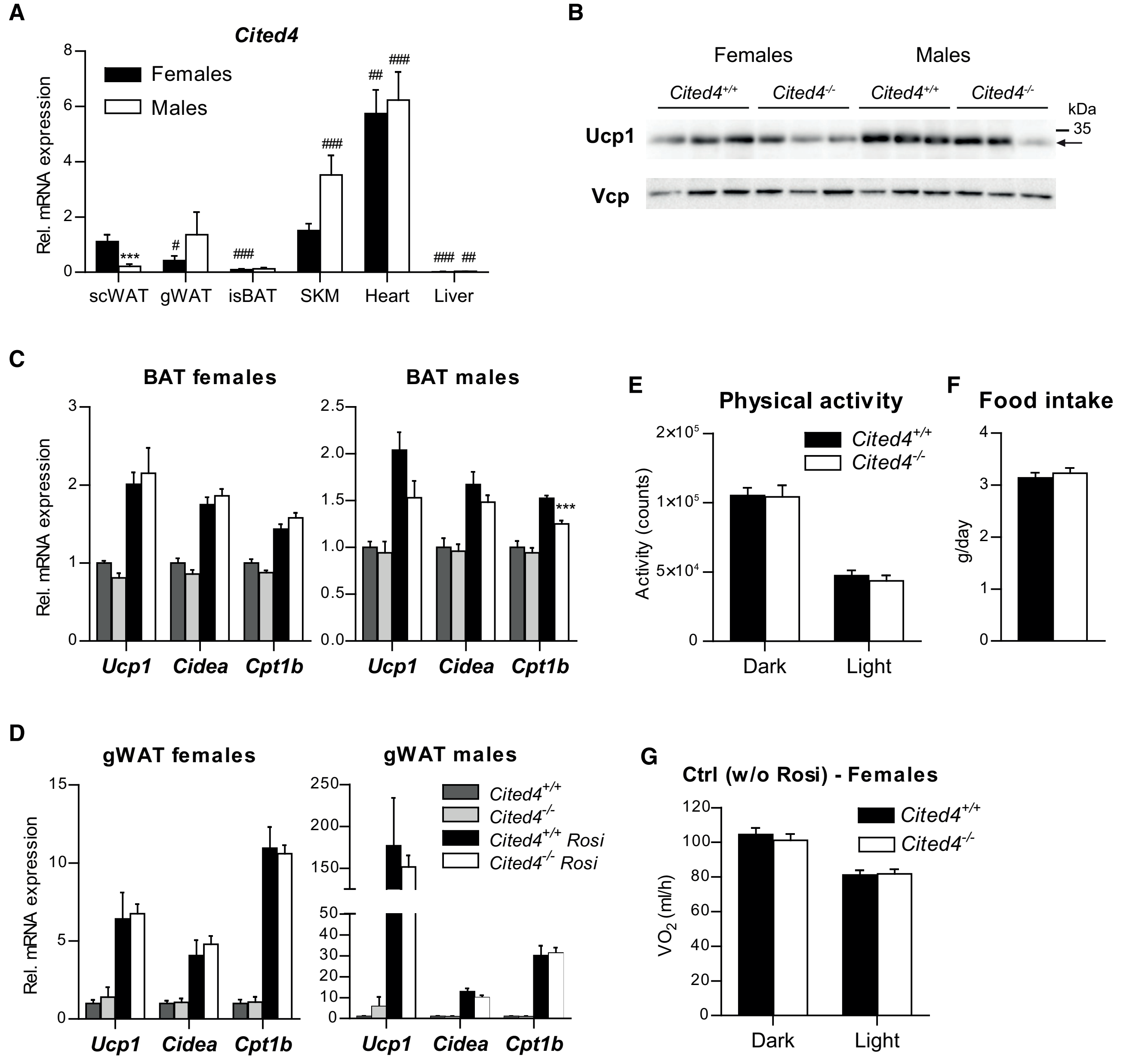

Figure EV3. *Cited4* deficiency specifically affects rosiglitazone-mediated thermogenic expression in subcutaneous fat.

A mRNA expression in wild-type mice without treatment, as determined by qRT–PCR (n = 6). scWAT: subcutaneous white adipose tissue; gWAT: gonadal WAT; isBAT: interscapular brown adipose tissue; SKM: gastrocnemius skeletal muscle. 2 × 2 ANOVA with Holm–Sidak posttests, $^{***}P$ = 0.0004 (female vs. male), $^{\#\#\#}P$ = 0.0004, $^{\#\#\#}P = 7 \times 10^{-6}$, $^{\#\#\#}P = 6 \times 10^{-9}$, $^{\#\#\#}P = 3 \times 10^{-10}$, (isBAT, SKM, heart, liver, respectively, vs. scWAT within indicated sex), $^{\#}P$ = 0.032 (females: gWAT vs. scWAT), $^{\#\#}P$ = 0.006 (females: Heart vs. scWAT), $^{\#\#}P$ = 0.006 (males: Liver vs. scWAT).

B Ucp1 expression in scWAT of mice fed a diet with 0.0075% Rosi or control diet for 2.5 weeks, determined by Western blot with VCP as loading control (n = 3, same samples as in Fig 4C and D).

C mRNA expression in BAT of mice treated as in (A), determined by qRT–PCR. t-test *Cited4*$^{-/-}$ vs. *Cited4*$^{+/+}$ (Rosi), females: n = 5/5/6/6, males: n = 5/4/5/5, $^{***}P$ = 0.0004.

D mRNA expression in gWAT of mice treated as in (A), determined by qRT–PCR. t-test *Cited4*$^{-/-}$ vs. *Cited4*$^{+/+}$ (Rosi), females: n = 5/5/6/6, males: n = 5/4/5/5.

E Three-day averages of total activity counts per day and mouse of female mice fed a diet with 0.0075% Rosi for 2.5 weeks, determined by Phenomaster (n = 9/10, t-test).

F Three-day averages of food intake per day and mouse of female mice shown in (E) (n = 9/10, t-test).

G Oxygen consumption rate of female mice fed control diet, determined by indirect calorimetry and adjusted for body weight by ANCOVA. Three-day averages of VO_2 were calculated for each mouse (n = 10). ANCOVA with Bonferroni's posttest (*Cited4*$^{-/-}$ vs. *Cited4*$^{+/+}$).

Data information: Data are presented as mean ± SEM.

Source data are available online for this figure.

© 2018 The Authors

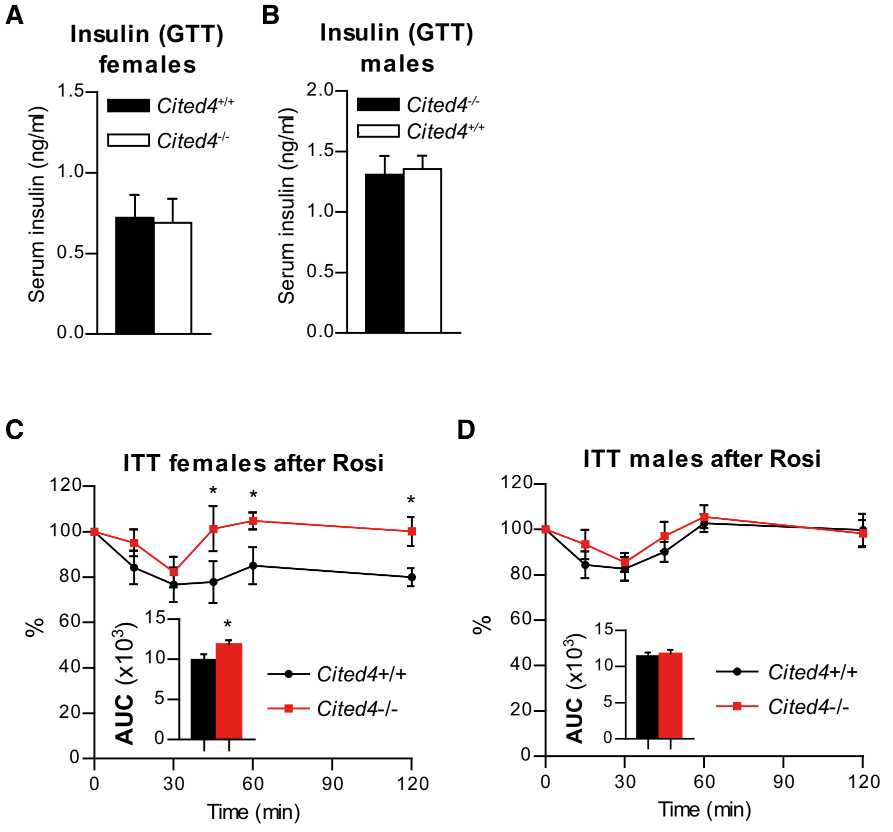

Figure EV4. Sex-specific involvement of *Cited4* insulin sensitization upon therapeutic rosiglitazone treatment.

A, B Serum insulin concentration 60 min after glucose injection in the GTT in mice after 11 weeks of HFD and 4 weeks of HFD + Rosi. *t*-test *Cited4*$^{-/-}$ vs. *Cited4*$^{+/+}$, (A) $n = 8$, (B) $n = 7/8$.

C Blood glucose during insulin tolerance test (ITT) with 1 U insulin per kg lean mass on female mice after 11 weeks of HFD and 4 weeks of HFD + Rosi, expressed as % of the 0-time point value ($n = 8$). *$P = 0.015$ (45 min), *$P = 0.039$ (60 min), *$P = 0.035$ (120 min) in repeated-measures 2 × 2 ANOVA with Holm–Sidak posttests; Inlet: AUC *t*-test *Cited4*$^{-/-}$ vs. *Cited4*$^{+/+}$ *$P = 0.041$.

D Blood glucose during ITT on male mice as in (C) ($n = 7/8$). Repeated-measures 2 × 2 ANOVA with Holm–Sidak posttests; Inlet: AUC *t*-test.

Data information: Data are presented as mean ± SEM.

© 2018 The Authors
