# Supplementary figures and images for: Cited4 is a sex‐biased mediator of the antidiabetic glitazone response in adipocyte progenitors
Source: EMBO Mol Med. 2018 Jul 4;10(8):e8613. doi: 10.15252/emmm.201708613 (PMC6079535; doi:10.15252/emmm.201708613)

Appendix Figure S1 - Panel B

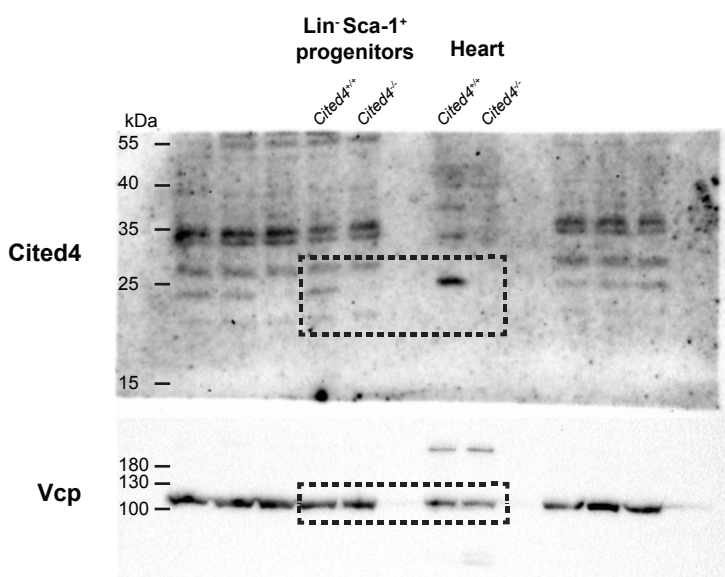

Appendix Figure S1 - Panel C

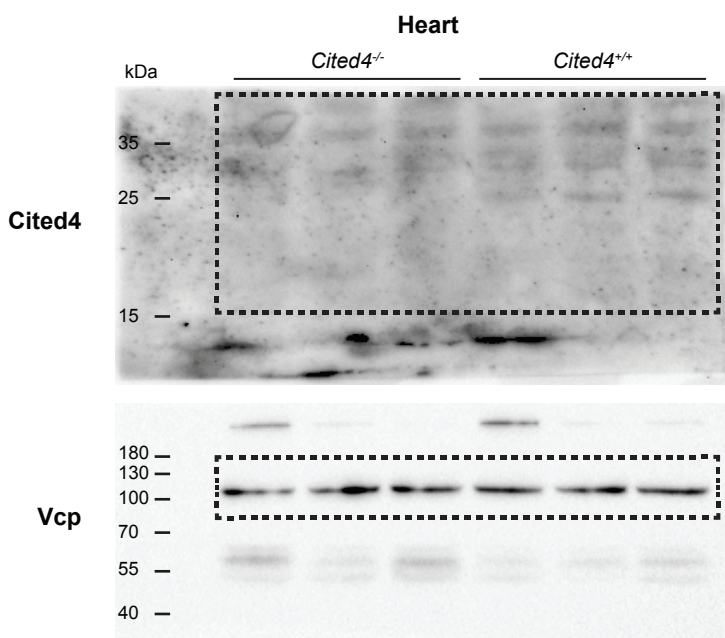

Appendix Figure S1 - Panel D

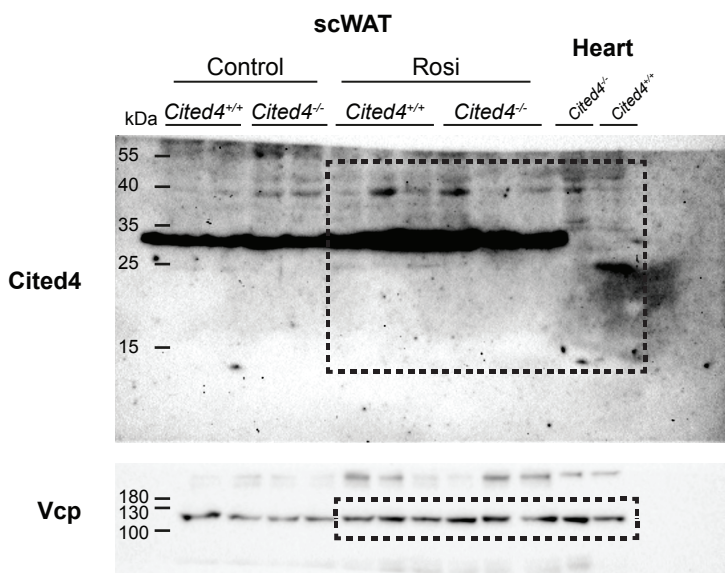

Supplement: Supplementary file 3 — Source Data for Expanded View and Appendix [file EMMM-10-e8613-s006.zip › emmm201708613-sup-0003-SDataAppendixFigS1B-D.pdf]

**Figure EV3 - Panel B**

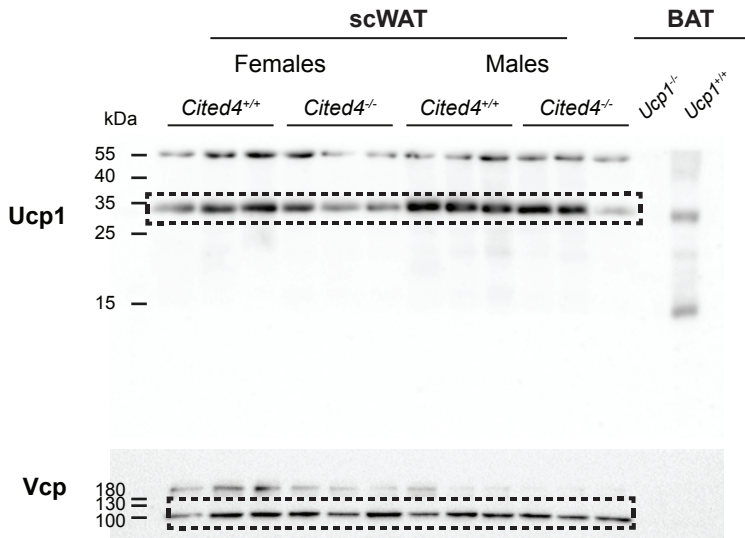

Supplement: Supplementary file 3 — Source Data for Expanded View and Appendix [file EMMM-10-e8613-s006.zip › emmm201708613-sup-0004-SDataFigEV3B.pdf]

Figure 2 - Panel D

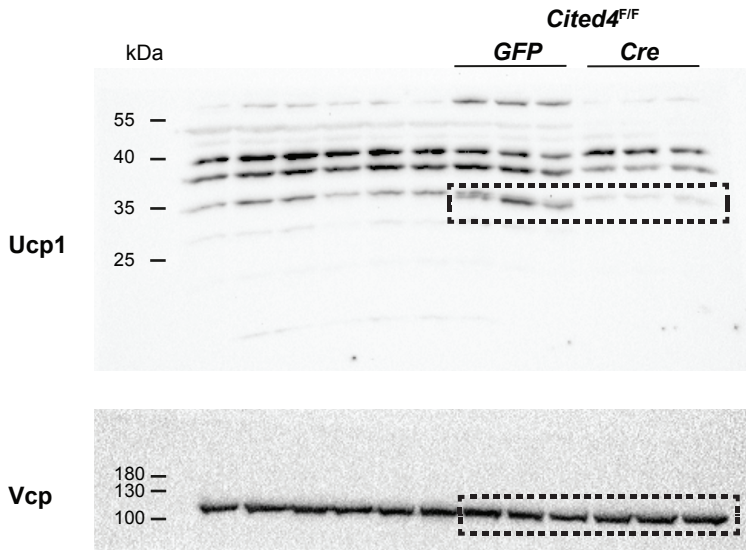

Supplement: Supplementary file 5 — Source Data for Figure 2 [file EMMM-10-e8613-s003.pdf]

**Figure 4 - Panel C**

**Females**

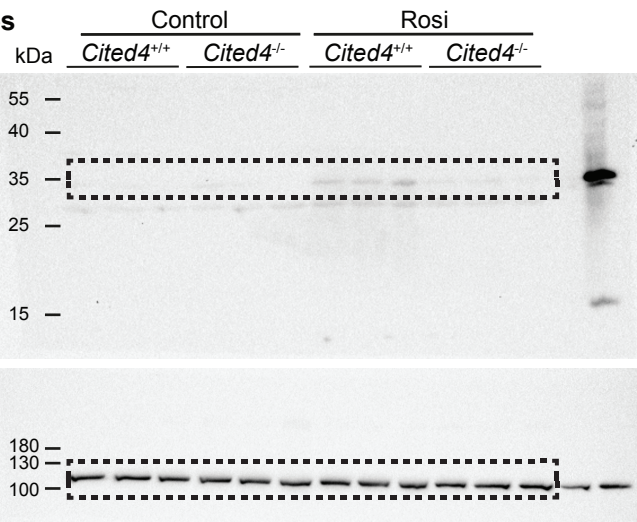

**Males**

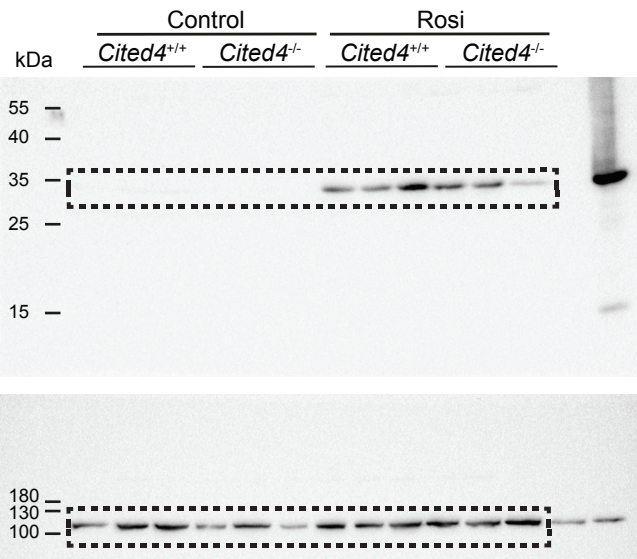

Supplement: Supplementary file 6 — Source Data for Figure 4 [file EMMM-10-e8613-s004.pdf]

**Figure 6 - Panel C**

**Females**

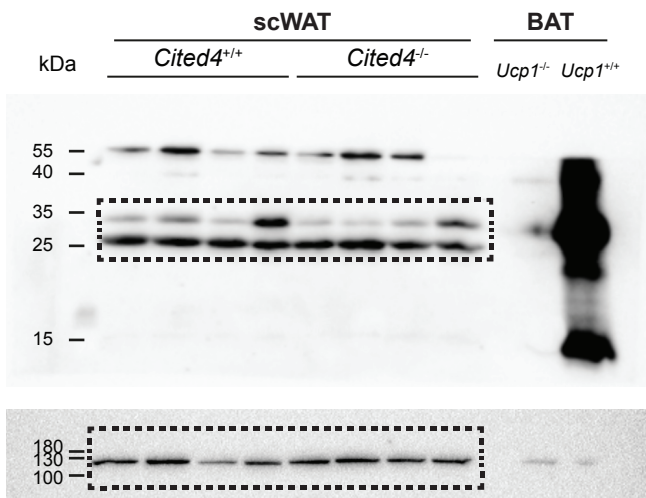

**Males**

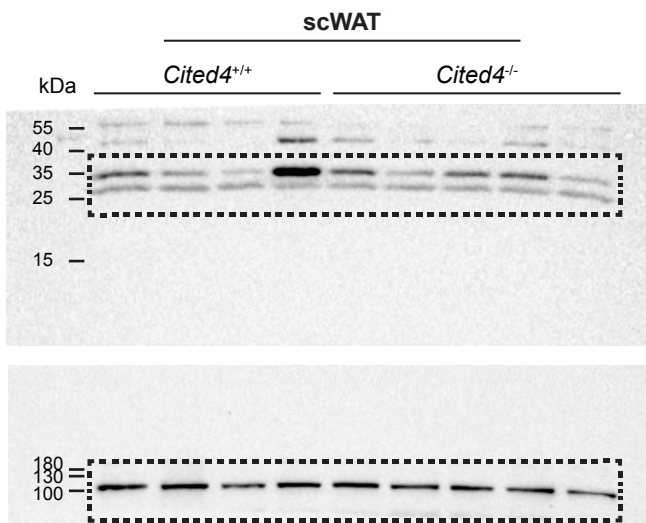

Supplement: Supplementary file 7 — Source Data for Figure 6 [file EMMM-10-e8613-s005.pdf]
